# Impact of acid and laser etching of enamel on microleakage in different adhesive systems

**DOI:** 10.1007/s10103-024-04120-0

**Published:** 2024-07-15

**Authors:** Sevim Atilan Yavuz, Ayse Tugba Erturk Avunduk, Ozcan Karatas, Nazire Nurdan Çakır Kılınç, Ebru Delikan

**Affiliations:** 1 Faculty of Dentistry, Department of Restorative Dentistry, Mersin University, Mersin, Türkiye; 2 Faculty of Dentistry, Department of Restorative Dentistry, Nuh Naci Yazgan University, Kayseri, Türkiye; 3 Faculty of Dentistry, Department of Pediatric Dentistry, Nuh Naci Yazgan University, Kayseri, Türkiye

**Keywords:** Adhesive systems, Er,Cr: YSGG laser, Microleakage, Selective etching

## Abstract

This study aimed to evaluate the microleakage of light-cured and self-cured adhesives on enamel surfaces selectively etched with Er, Cr: YSGG laser or 35% phosphoric acid. A total of 60 class V cavities were prepared 1 mm above the cemento-enamel junction (CEJ). The specimens were randomly divided into six groups. Group 1: Clearfil SE Bond with no conditioning, Group 2: Tokuyama Universal Bond with no conditioning, Group 3: Clearfil SE Bond conditioned with 35% phosphoric acid, Group 4: Tokuyama Universal Bond conditioned with 35% phosphoric acid, Group 5: Clearfil SE Bond conditioned with Er, Cr: YSGG laser and Group 6: Tokuyama Universal Bond conditioned with Er, Cr: YSGG laser. Microleakage was evaluated qualitatively (visually) and quantitatively (ImageJ). The data were analyzed using IBM SPSS V23 and submitted to Kruskal–Wallis and Wilcoxon tests. The significance level was set at *p* < 0.05. In all evaluation methods, the microleakage scores exhibit significant differences (p*<0.001). Group 1 and Group 3 exhibited similar and lower microleakage values than the Group 5. In the occlusal margin, the microleakage values were similar in Group 2, Group 4, and Group 6, whereas in the gingival margin Group 4 showed significantly lower leakage compared to Group 2. Regardless of the etching protocols and adhesive systems used, less microleakage was observed on the occlusal surface than on the gingival surface. Phosphoric acid etching provides better results than laser etching for enamel surface treatment on both occlusal and gingival surfaces.

**Supplementary Information:**

The online version contains supplementary material available at 10.1007/s10103-024-04120-0.

## Introduction

As awareness of individual health increases, patient expectations in dental treatments are evolving, leading to a growing demand for aesthetic restoration in posterior teeth. Resin composite materials, known for their advancing abrasion resistance, ease of application, and aesthetic properties, have emerged as the preferred choice for restorative procedures [1]. However, a significant drawback of resin composites is their susceptibility to polymerization shrinkage [2]. When the shrinkage exceeds the bond strength between the composite and cavity walls, separation occurs, resulting in gap formation. This gap permits the infiltration of oral fluids and bacteria, leading to microleakage—a substantial concern for the long-term clinical performance of composite resin restorations [3]. To mitigate these issues, altering enamel surface properties through treatments like phosphoric acid or laser interventions has been proposed for enhancing the clinical adhesion success of composite resins [4, 5].

Efficient bonding poses a common challenge in clinical practice, particularly in class V cavities [6]. Despite significant efforts to develop adhesive systems for strong and stable dentin bonds, conflicting results persist in the literature regarding occlusal and gingival enamel margin microleakage. Fathpour et al. [7] noted differences in leakage at the gingival margin between two-step self-etch and universal adhesive systems, irrespective of the etching mode. Conversely, another in-vitro study reported no distinction between two-step self-etch and universal adhesive systems [8]. Lasers have recently been used to improve adhesion in addition to the aforementioned procedures. The utilization of lasers in restorative dentistry, especially Erbium, Chromium: Yttrium, Scandium, Gallium, Garnet (Er; Cr: YSGG) lasers, is gaining popularity for minimally invasive restoration preparation and enhanced bonding to dental tissues [9]. While existing literature predominantly explores the cavity preparation and disinfection effectiveness of Er; Cr: YSGG lasers [10, 11], limited documentation exists on their etching impact on class V cavity margins.

This study aims to assess the microleakage effectiveness of light-cured two-step self-etch and self-cured universal adhesive systems in class V cavities selectively etched with phosphoric acid or Er, Cr: YSGG lasers. The null hypotheses tested are that there is no difference between selective acid etching and laser etching methods in terms of microleakage in class V occlusal and gingival enamel margins, and there is no difference among adhesive systems concerning microleakage in class V occlusal and gingival enamel margins.

## Materials and methods

**Fig. 1:**
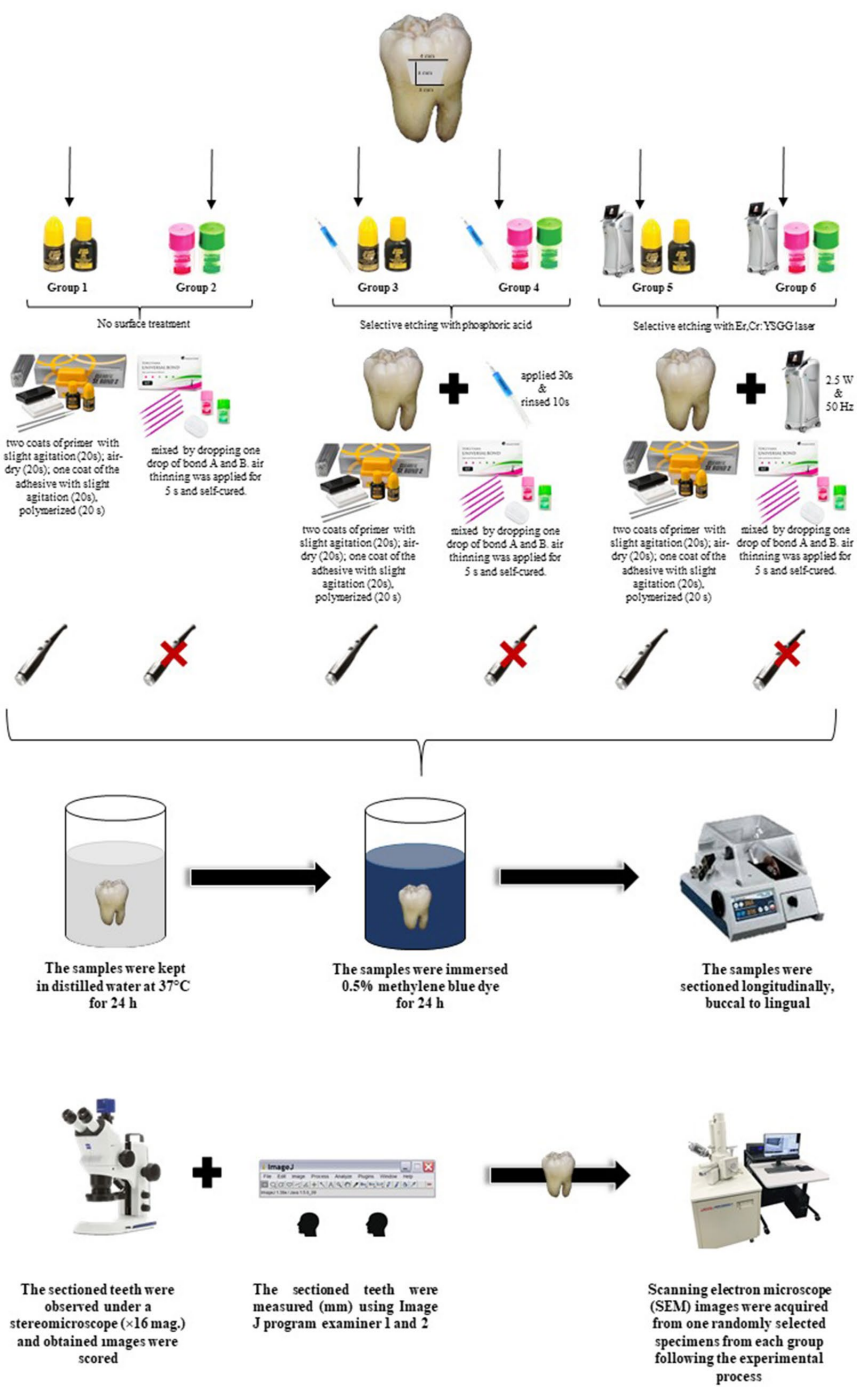
Flowchart of the study

This study was approved by the Ethics Committee of the Nuh Naci Yazgan University. The present investigation was conducted to examine the effectiveness on microleakage of light-cured two steps self-etch (Clearfil SE Bond) and self-cured universal adhesive systems (Tokuyama Universal Bond II™) in class V cavities that were selective etched with phosphoric acid or Er, Cr: YSGG lasers. The flowchart of experimental process of the study are shown in Fig. 1.

### Specimens preparation

The determination of the sample size was conducted through a power analysis using the G-Power package program (Version 3.1.9.4, Heinrich Heine University of Düsseldorf, Düsseldorf, Germany). An assumed statistical power of 80% and a significance level of 0.05 were employed. Initially, an estimated total sample size of 28 specimens was determined. To address potential dropout rates the sample size was subsequently augmented, culminating in a final aggregate of 30 subjects for the study [12].

Thirty extracted sound human third molar teeth with no caries, cracks and restorations were used in the study. Before use, the calculus, residual soft tissues and periodontal fibers on the root surface were meticulously removed with a scaler (Hu–Friedy Mfg. Co., Chicago, USA) after that the teeth were polished with the slurry of pumice and teeth were stored in 0.01% thymol solution in a dark environment. Class V cavities were prepared in the buccal and lingual surfaces of each tooth (total of 60 cavities in 30 teeth) with diamond fissure bur (Komet Dental, Brasseler GmbH and Co., Lemgo, Germany). A new bur was used following every five cavities for cutting efficiency. The cavity dimensions were prepared as “occlusal wall 4 mm (± 0.2 mm) and gingival wall 3 mm (± 0.2 mm) mesiodistal width, 3 mm (± 0.2 mm) occlusal-gingival height and 2 mm (± 0.2 mm) depth” under water-cooling and cavity dimensions were measured with a periodontal probe. The gingival margin was placed 1 mm above the cemento-enamel junction (CEJ). All preparations up to the experimental process of the study were performed by the same operator (E. D.). The accuracy of prepared cavities on specimens was checked by blinded operators (O. K. and N. N. Ç. K.).

### Experimental groups

The specimens were selected by a simple random method without replacement using an online randomization software (randomizer.org) and were divided into six groups (Fig. 1). The groups were classified according to type of adhesive systems (Clearfil SE Bond and Tokuyama Universal Bond™) and selective etching protocols [35% phosphoric acid (Ultra-Etch™, Ultradent Products, Inc) and Er, Cr: YSGG laser (Waterlase iPlus, Biolase, Waterlase)]. The chemical composition, LOT numbers, and manufacturer of the restorative materials and adhesive systems are presented in Table 1. The type of the employed adhesive systems groups were as follows:

- Group 1 (no conditioning + light-cured SE): Clearfil SE Bond (Kuraray Medical LTD, Osaka, Japan) was applied to the cavity surface [air-dry the dentin surface; two coats of primer with slight agitation (20s); air-dry (20s at 20 cm); one coat of the adhesive with slight agitation (20s)] and polymerized by the light-emitting diode (LED, D-Light Pro, GC, Japan) with an irradiance of 1200 mW/cm^2^, following the manufacturer’s recommendations for 20 s without any enamel conditioning.
- Group 2 (no conditioning + self-cured Universal): Tokuyama Universal Bond™ adhesive bottle system (TokuyamaDental, Tokyo, Japan) was mixed by dropping one drop of bottles A and B. The adhesive was scrubbed to the cavity surface with a disposable applicator and agitation for 20 s. Then, air thinning was applied for 5 s and self-cured without any enamel conditioning.
- Group 3 (selective acid etching + light-cured SE): Enamel edges were etched with 35% phosphoric acid gel (Ultra-Etch™, Ultradent Products, Inc) for 30 s and rinsed 10 s. Clearfil SE Bond was applied to the cavity surface in the same application mode as *Group 1*.
- Group 4 (selective acid etching + self-cured Universal): Enamel edges were etched with 35% phosphoric acid gel (Ultra-Etch™, Ultradent Products, Inc) for 30 s and rinsed 10 s. Tokuyama Universal Bond™ was applied to the cavity surface in the same application mode as *Group 2.*
- Group 5 (laser + light-cured SE): Enamel edges were selectively etched by Er, Cr: YSGG laser (Biolase, Waterlase, USA) with the following parameters were used: 2,780 nm wavelength, 20 Hz repetition rate, 140 µs pulse duration, 1.25 W output power. The focus mode employed a sapphire-tipped apparatus, featuring a diameter of 600 μm, positioned at a focal distance of 1–2 mm. The cooling system parameters adhered to the manufacturer’s instructions, comprising a composition of 60% water and 70% air. The laser parameters were not changed during the entire conditioning. Clearfil SE Bond was applied to the cavity surface in the same application mode as *Group 1*.
- Group 6 (laser + self-cured Universal): Enamel edges were selectively etched by Er, Cr: YSGG laser with the same application protocol as Group 5. Tokuyama Universal Bond™ was applied to the cavity surface in the same application mode as *Group 2.*

**Table 1:** Chemical composition, batch numbers, and instructions for use of the restorative materials

Product	Type	Composition	Manufacturer	LOT (Batch) Number
Clearfil SE Bond	Two steps self-etch adhesive system	Primer: MDP, HEMA, hydrophilic aliphatic dimethacrylate, dl-camphorquinone and water Bond: MDP, HEMA, Bis-GMA, hydrophilic aliphatic dimethacrylate, dl-camphorquinone, initiators and accelerators silanated colloidal silica pH: 2	Kuraray Europe GmbH, Düsseldorf, Germany	000483
Tokuyama Universal Bond	One step universal adhesive system	Bond A: acetone, phosphoric acid monomer, Bis-GMA, TEGDMA, HEMA and MTU-6 Bond B: acetone, isopropanol, water, borate catalyst, peroxide and silane coupling agent pH: 2.2	Tokuyama Dental, Tokyo, Japan	004E42
Ultra-Etch	Etchant	35% phosphoric acid, water, cobalt aluminate blue spinel, glycol, siloxane	Ultra-Etch™, Ultradent Products, Inc.	BFDJ9
G-aenial Posterior Composite	Microhybrid composite resin	UDMA and dimethacrylate co-monomer	GC Corp., Tokyo, Japan	1,709,223

*MDP *10-Methacryloyloxydecyl dihydrogen phosphate, *HEMA *2-Hydroxyethyl methacrylate, *Bis-GMA *Bisphenol A diglycidylmethacrylate, *TEGDMA *Triethylene glycol dimethacrylate

### Restoration procedures

Following the adhesive systems procedures, each cavities of 2 mm depth were restored with a micro-hybrid G-ænial Posterior (GC Corp., Tokyo, Japan) composite resin in a single layer and light cured for 20 s using the LED (D-Light Pro, GC, Japan) with an irradiance of 1200 mW/cm^2^. A calibrated radiometer (Blast LED Light Meter, First Medica, Greensboro, NC, USA) was used to verify the irradiance of the light-curing unit. All procedures and curing times were performed according to the manufacturer’s instructions. The restorations were finished with fine-grit finishing diamond burs (Komet Dental, Brasseler GmbH and Co., Lemgo, Germany), then polished with sandpaper disks (Sof-Lex, 3 M ESPE). The specimens were kept in distilled water at 37 °C for 24 h for post-polymerisation. All specimens were aged in a thermocycler (Esetron, Turkey) in distilled water for 2,000 cycles in a 5–55 ^0^C water bath. The duration of the thermocycler was set as 30 s, and the transfer time was 5 s. Then, the root apex of specimens was sealed with a resin composite and the specimens were dried with absorbent paper tissue and air and then two coats of clear nail varnish (Flormar, Milan, Italy) were applied to the tooth within 1 mm of the restoration margins to avoid penetration of the dye from the pores and restoration margins. The specimens were separately immersed in freshly prepared 2% methylene blue solution (Sigma-Aldrich Co., St Louis, MO) for 24 h and then embedded in a mold to obtain an acrylic resin.

### Evaluation of microleakage with light microscopy

To detect the microleakage, each tooth were sectioned longitudinally, buccal to lingual, with a water-cooling, slow-speed, diamond saw (Isomed, Buehler Ltd, Lake Bluff, IL, USA). After thermocycling, both sides of all specimen sections were ground using silicon carbide abrasive paper with grit sizes of 400, 600, and 1,200 (Leco1 VP 100, Leco Instrumente GmbH, Germany). Subsequently, a 35% phosphoric acid gel (Ultra-EtchTM, Ultradent Products, Inc.) was applied for 60 s, followed by the application of a 2% sodium hypochlorite solution for 120 s to remove the smear layer from the section surfaces. The cut surfaces of the tooth-restoration interface were examined at the occlusal and gingival margins using a light microscope (Zeıss Opmi pıco, Carl Zeıss Meditec AG, Germany) at ×16 magnification. The penetration values of methylene blue were evaluated in two methods:

a using the open-source image analysis software program (ImageJ, V.1.42, National Institutes of Health, Bethesda, MD) to record the penetrations in the “mm” scale, and measurements were taken for the overall length of the tooth-restoration interface, as well as the length of the stained interface.
b examining dye penetration at both enamel and dentin edges visually. The dye penetration of each specimen was evaluated using the scoring system described in Table 2 [13]. Scores were assigned to each individual section with a millimeter scale (± 0.5 mm) built into the microscope optic.

**Table 2:** Score system to quantify microleakage at the tooth-restoration interfaces (when the cavity depth was 2 mm)

Score	Microleakage
0	No dye penetration (0 mm)
1	Dye penetration of up to 1/3 of the cavity wall (0– 0,66 mm)
2	Dye penetration of more than 1/3, but less than 2/3, of the cavity wall (0,66– 1,32 mm)
3	Dye penetration of more than 2/3, or to the full extent of the cavity wall (> 1,32 mm)

**Fig. 2:**
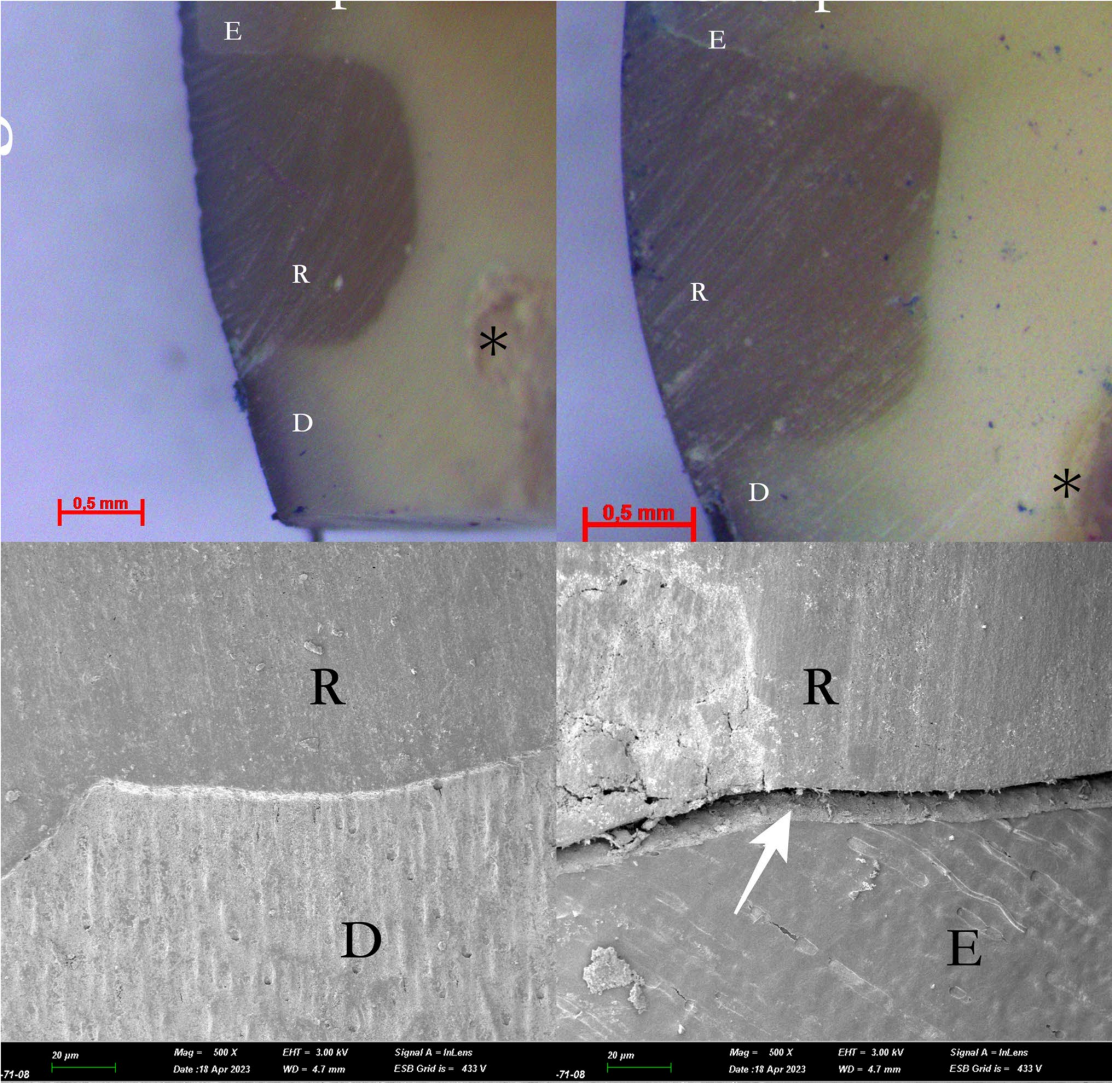
The light microscope and SEM images of the no-conditioning groups (Group 1 and 2) *: pulp, D: Dentin, E: Enamel, R: Restoration, White narrow: Gap formation

**Fig. 3:**
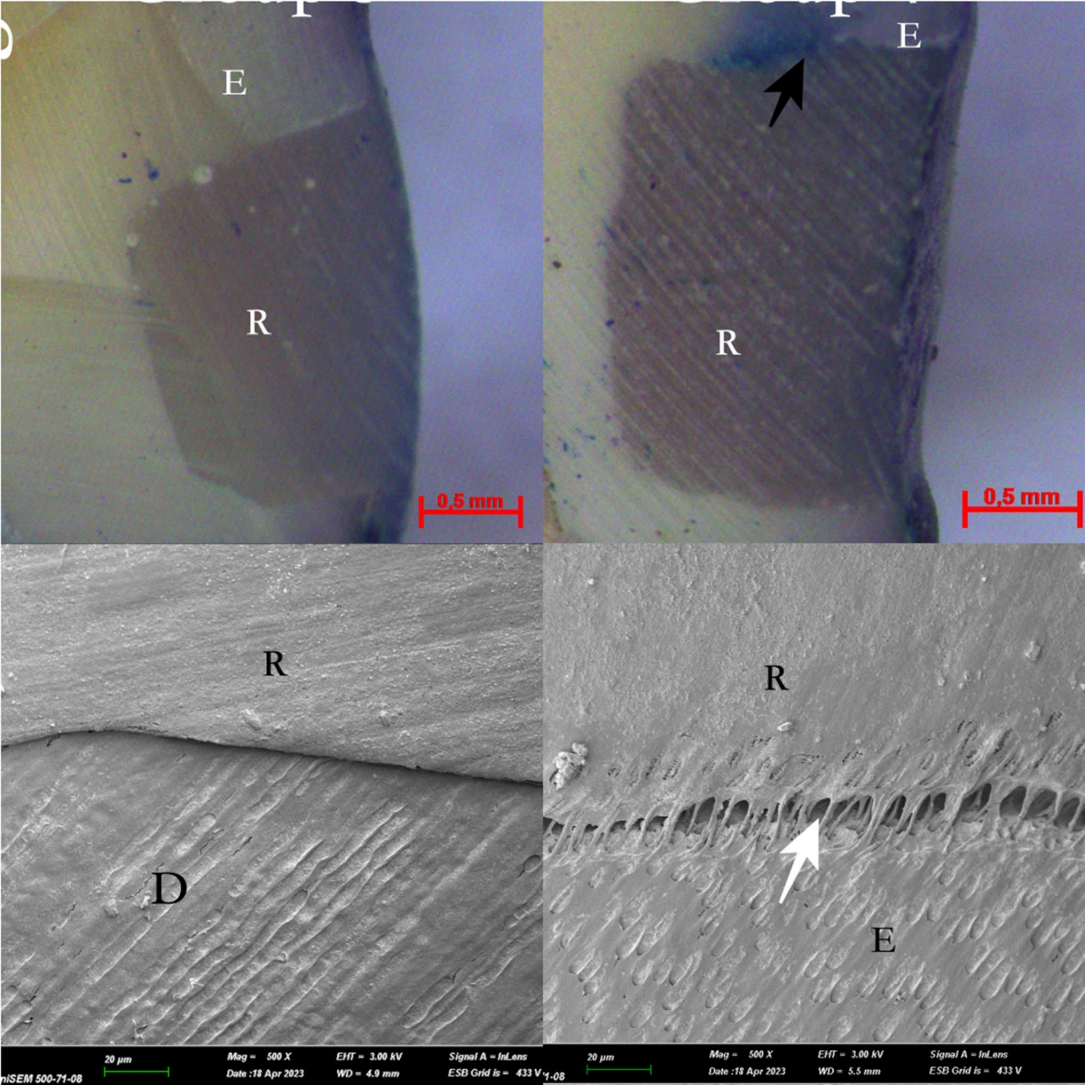
The light microscope and SEM images of the selective acid etching groups (Group 3 and 4) *: pulp, D: Dentin, E: Enamel, R: Restoration, White narrow: Gap formation, Black Narrow: Methylen blue dye penetration

**Fig. 4:**
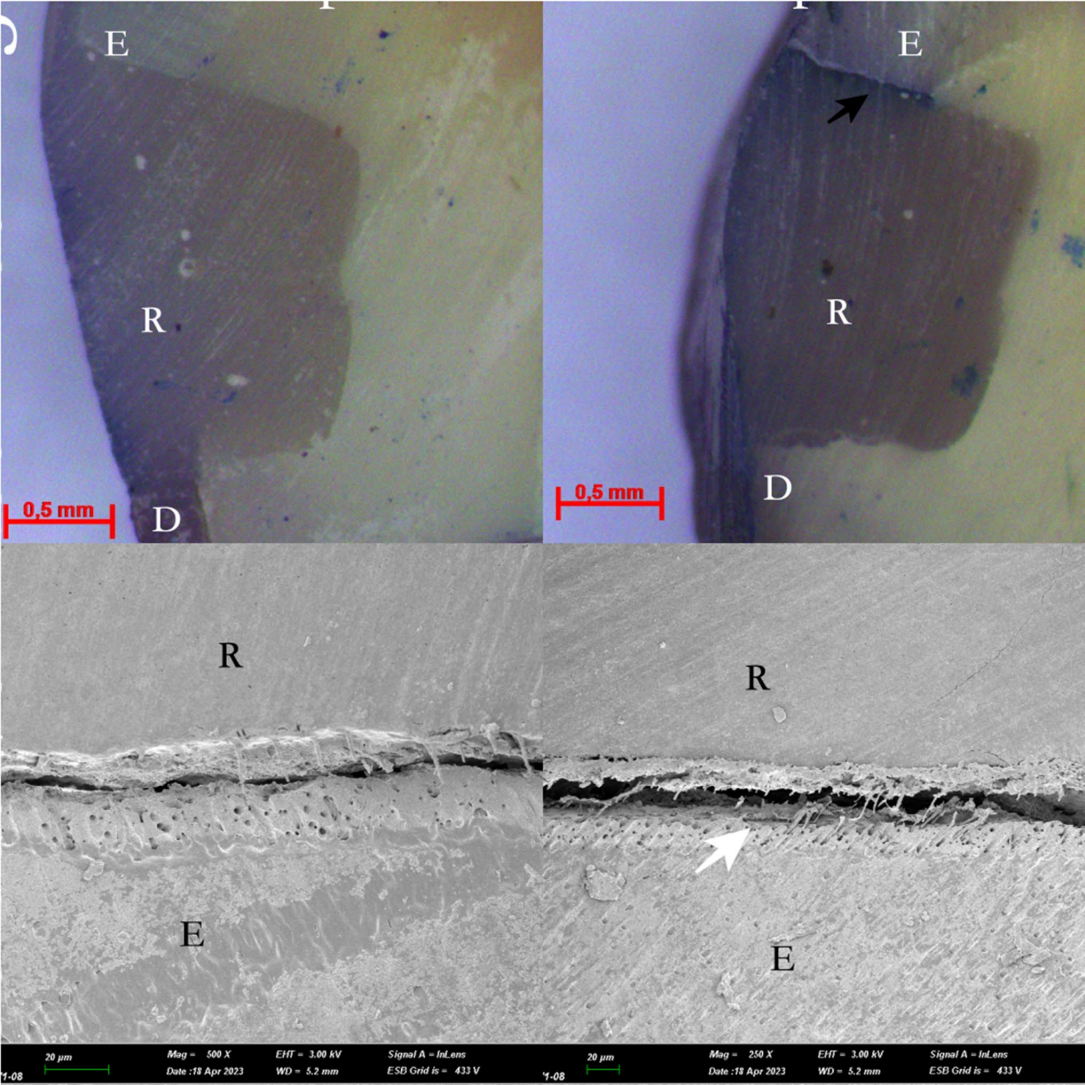
The light microscope and SEM images of the selective laser etching groups (Group 5 and 6) *: pulp, D: Dentin, E: Enamel, R: Restoration, White narrow: Gap formation, Black Narrow: Methylen blue dye penetration

Microleakage assessments at both methods were conducted independently by two experienced and blinded investigators (S. A.Y., A.T. E. A) in the occlusal and gingival enamel margins. Any scoring discrepancies between the investigators were thoroughly discussed until a consensus was reached, resulting in a final agreed-upon score. The light microscope images are presented in Figs. 2, 3 and 4.

### Evaluation of microleakage with Scanning Electron Microscopy (SEM)

Scanning electron microscope (SEM) images were acquired from one randomly selected specimens from each group following the experimental process. Following dehydration in an aqueous ethanol solution, the specimens underwent palladium coating using the ion plating unit (Polaron SC500 sputter coater, FISONS Instrument, UK). Subsequently, careful observation of the specimens was conducted using a SEM (JSM-5600LV, JEOL, Tokyo, Japan) at a magnification of x16. In case of any conflicts in scores, consensus was reached between observers through discussion and mutual agreement. The SEM images are presented in Figs. 2, 3 and 4.

### Statistical analysis

Statistical analysis was obtained by using IBM SPSS V26 (SPSS Inc., Chicago, IL, USA) software. Compliance with normal distribution was examined with the Shapiro-Wilk Test. Kruskal Wallis Test was used to examine the differences between microleakage scores that did not comply with normal distribution according to groups, and multiple comparisons were performed with Dunn Test. Paired Two-Sample t Test was used to compare occlusal and cervical measurements with normal distribution in each group, and Wilcoxon Test was used to compare those with non-normal distribution. Intraclass Correlation Coefficient (ICC) was used to examine interobserver agreement. Analysis results were presented as median (minimum – maximum). The significance level was taken as *p* < 0.050.

## Results

### Comparison of microleakage values between treatment groups

As shown in Table 3, in all evaluation methods (Image J, Examiner 1, and Examiner 2), the microleakage scores in the occlusal and gingival margins exhibit significant differences (p * < 0.001).

**Table 3:** Results of comparison of microleakage scores in dentin (cervical) and enamel (occlusal) sites within the groups

		Treatment Groups							
		No-conditioning		Selective Acid Etching		Selective Laser Etching			
Assesment methods	Tooth region	Group 1	Group 2	Group 3	Group 4	Group 5	Group 6	Test İs.	p*
Image J	Occlusal	0.16 ± 0.25	0.27 ± 0.49	0.08 ± 0.18	0.27 ± 0.27	0.49 ± 0.38	0.21 ± 0.17	37.84	< 0.001*
		0.00 (0.00 – 0.81) ^ab^	0.00 (0.00 – 1.35) ^bd^	0.00 (0.00 – 0.65) ^a^	0.26 (0.00 – 0.80) ^bd^	0.46 (0.00 - 1.45)^cd^	0.21 (0.00 – 0.60) ^abd^		
	Cervical	0.47 ± 0.56	0.70 ± 0.44	0.33 ± 0.49	0.27 ± 0.30	0.95 ± 0.33	0.54 ± 0.28	30.19	< 0.001*
		0.16 (0.00 – 1.44) ^a^	0.77 (0.00 – 1.47) ^b^	0.00 (0.00 – 1.14) ^a^	0.18 (0.00 – 0.97) ^a^	0.90 (0.33 – 1.54) ^b^	0.62 (0.00 – 0.93) ^ab^		
Test İst.		–1.804	–2.487	–1.836	–0.199	–3.98	–3.136		
p**/***		0.071**	0.013**	0.066**	0.842**	0.001***	0.002**		
Examiner 1	Occlusal	0.42 ± 0.51	0.68 ± 1.16	0.32 ± 0.67	0.84 ± 0.69	1.32 ± 0.95	0.84 ± 0.50	27.23	< 0.001*
		0.00 (0.00 – 1.00) ^ab^	0.00 (0.00 – 3.00) ^b^	0.00 (0.00 – 2.00) ^a^	1.00 (0.00 – 2.00) ^bc^	1.00 (0.00 – 3.00) ^c^	1.00 (0.00 – 2.00) ^bc^		
	Cervical	1.21 ± 1.27	1.26 ± 0.81	0.84 ± 1.26	0.95 ± 1.03	2.47 ± 0.61	1.53 ± 0.70	29.58	< 0.001*
		1.00 (0.00 – 3.00) ^ab^	1.00 (0.00 – 3.00) ^ab^	0.00 (0.00 – 3.00) ^ab^	1.00 (0.00 – 3.00) ^ab^	3.00 (1.00 – 3.00) ^c^	2.00 (0.00 – 2.00) ^bc^		
Test İst.		–2.218	–1.6	–1.628	–0.318	–3.054	–2.968		
p**		0.027**	0.110**	0.103**	0.751**	0.002**	0.003**		
Examiner 2	Occlusal	0.47 ± 0.70	0.74 ± 1.24	0.32 ± 0.67	0.68 ± 0.82	1.58 ± 1.02	1.05 ± 0.62	34.66	< 0.001*
		0.00 (0.00 – 2.00) ^ab^	0.00 (0.00 – 3.00) ^ab^	0.00 (0.00 – 2.00) ^a^	0.00 (0.00 – 2.00) ^ab^	2.00 (0.00 – 3.00) ^c^	1.00 (0.00 – 2.00) ^bc^		
	Cervical	1.21 ± 1.36	1.74 ± 0.99	0.84 ± 1.26	1.32 ± 1.16	2.89 ± 0.32	2.26 ± 1.10	36.16	< 0.001*
		1.00 (0.00 – 3.00) ^ab^	2.00 (0.00 – 3.00) ^b^	0.00 (0.00 – 3.00) ^a^	1.00 (0.00 – 3.00) ^ab^	3.00 (2.00 – 3.00) ^c^	3.00 (0.00 – 3.00) ^bc^		
Test İst.		–1.969	–2.364	–1.628	–2.029	–3.36	–3.348		
p**		0.049**	0.018**	0.103**	0.042**	0.001**	0.001**		

*Kruskal Wallis Test; Mean ± standard deviation; Median (minimum-maximum); a-d: No difference exists between groups with the same letter

**Wilcoxon Test; ***Paired Two Sample t Test; Mean ± standard deviation; Median (minimum-maximum)

Within the Image J assessment, Group 1 showed similar microleakage values in the occlusal and significantly lower microleakage values in the gingival margins compared to Group 2. In both margins (occlusal and gingival), Group 1 and Group 3 exhibited similar and lower microleakage values than Group 5. In the occlusal margin, the microleakage values were similar in Group 2, Group 4, and Group 6, whereas, in the gingival margin, Group 4 showed significantly lower leakage compared to Group 2.

According to Examiner 1’s assessment, in both margins, Group 1 demonstrated similar microleakage values to Group 2. The Group 1 and Group 3 groups exhibited similar low microleakage values, while Group 5 showed higher microleakage than these two groups. Microleakage values were similar in Group 2, Group 4, and Group 6. Group 5 exhibited significantly higher microleakage compared to Group 1, Group 2, and Group 3. Two different laser groups, however, showed similar microleakage values.

According to Examiner 2’s assessment, there was no significant difference in microleakage values between Group 1 and Group 2 in both occlusal and gingival margins. Group 1 and Group 3 exhibited similar low microleakage values, while Group 5 showed higher microleakage than these two groups. The microleakage values were similar in Group 2, Group 4, and Group 6. The highest and similar microleakage was observed in the two laser groups.

### Comparison of microleakage values within assessment groups

As shown in Table 3, the microleakage values in the occlusal and gingival margins of Group 1 exhibited significant differences in the assessments of Examiner 1 and Examiner 2 (*p* = 0.027 and *p* = 0.049, respectively). According to these assessments, microleakage in the occlusal margin was lower than in the gingival margin. In Group 2, the microleakage values in the occlusal and gingival margins showed significant differences in the evaluations of Image J and Examiner 2 (*p* = 0.013 and *p* = 0.018, respectively). According to these assessments, microleakage in the occlusal margin was lower than in the gingival margin. For Group 3, no significant differences were observed among the assessments (Image J, Examiner 1, Examiner 2) (*p* > 0.05). In Group 4, lower leakage was observed in the occlusal margin compared to the gingival margin only in the assessment of Examiner 2 (*p* = 0.042). In Group 5 and Group 6, microleakage in the occlusal margin was significantly lower than in the gingival margin in all assessment methods.

### Correlation between assessment methods

A high level of statistical agreement was observed between Examiner 1 and Examiner 2 in the assessment of occlusal and gingival microleakage measurements, as indicated by the intraclass correlation coefficients (ICC) of 0.907 (*p* < 0.001) and 0.954 (*p* < 0.001) respectively. Furthermore, a statistically significant and commendable level of agreement was attained between the observations of Image J and Examiner 1 for both occlusal and gingival microleakage measurements, with ICC values of 0.794 (*p* < 0.001) and 0.781 (*p* < 0.001) respectively. Similarly, there was a noteworthy statistical agreement between Image J and Examiner 2 observers for occlusal and gingival microleakage assessments, with ICC values of 0.725 (*p* < 0.001) and 0.713 (*p* < 0.001) respectively. These findings underscore the reliability and consistency of the assessments performed by the different observers and measurement methods in the evaluation of microleakage.

## Discussion

In light of advancements in dental materials, the integration of composite resin restorations into clinical practices has been progressively realized [13]. Nevertheless, a primary drawback inherent to composite resin materials is polymerization shrinkage, which can engender a marginal gap at the interface between the tooth and the restoration, thereby affecting the long-term success of restorations [14]. This phenomenon, characterized by the infiltration of bacteria, fluids, molecules, or ions through these marginal gaps, is termed microleakage and is widely recognized as a pivotal factor contributing to the development of secondary caries and pulpal infections [15]. From this viewpoint, the current experimental study was designed to evaluate the microleakage of light-cured and self-cured adhesives on enamel surfaces selectively etched with Er, Cr: YSGG laser or 35% phosphoric acid.

Microleakage assumes heightened importance in class V cavities, which were chosen as the focus of the current study due to their recognized suitability for evaluating bonding effectiveness across several dimensions [13]. Since there is a significant decrease in enamel thickness towards the CEJ, the enamel in the gingival margins of Class V cavities is considerably thinner than in the occlusal margin. Consequently, this condition renders the gingival margins susceptible to marginal leakage in comparison to the occlusal margins. [16]. The occurrence of leakage at the gingival margins of Class V resin composite restorations is a commonly observed phenomenon, therefore the ability of adhesive systems to hybridize the hard tissues should be questioned [17]. Enamel, particularly at the gingival margin, is usually thin, aprismatic, and exhibits reduced bonding affinity with resin materials [18]. During the polymerization process, monomer molecules within resin-based materials undergo displacement as they form covalent bonds, resulting in a contraction of the inter-monomer distance [19]. The resin composite contracts towards the more robust bond established at the occlusal margin, causing disengagement from the comparatively weaker bond at the gingival margin. The thicker cavity wall and organized prism structure present at the occlusal enamel margin facilitate sufficient bonding, thereby yielding restorations with reduced instances of leakage [17]. In the current investigation, the inconsistency in microleakage observed at the occlusal and gingival margins of the specimens may be attributed to these discrepancies in the polymerization shrinkage process. The different application modes of the tested adhesives yielded varying microleakage values within class V cavities subjected to selective etching with phosphoric acid or Er, Cr: YSGG lasers. Consequently, the first hypothesis posited in the present study, asserting the absence of disparities in microleakage between selective etching and laser etching methods, is hereby rejected. It is well-established that optimal adhesion to enamel and sealing efficacy is achieved through the micromechanical interlocking of resin with irregularities created by phosphoric acid on the enamel surface [20]. Concur with these results, less microleakage was observed in the Group 3 in both occlusal and gingival margins than in no-conditioned (Group 1 and 2) and laser groups (Group 5 and 6), in the present study. Similarly, Fattah et al. [15] reported that less microleakage was seen in acid-etched cavities. Despite many adhesive systems developed to enhance bonding, microleakage remains to be a significant clinical concern due to the thinner enamel thickness at the gingival margins of restorations [21]. In the current study, it was observed that there was a significant difference in terms of microleakage in the occlusal and gingival margins in the laser-conditioned groups (Group 5 and 6) and in one of the no-conditioned group (Group 2). Fathpour et al. [7] have consistently demonstrated that using phosphoric acid etching results in decreased microleakage at enamel margins. The findings of the present study were in line with the study of Fathpour et al. [7], lower microleakage values were obtained in Clearfil SE Bond in selective-etch mode compared to self-etch mode.

As a result of exposure of dental tissue to the erbium laser, the interstitial water in the target tissue absorbs photonic energy. As a result of molecules emerge in the vapor phase and cause fragmentation and irregularities in the tooth tissue as a result of micro explosions that occur when the vapor pressure exceeds the tissue strength [22]. Since laser applications are effective on the amount of water in the target tissue, studies have found that erbium laser applications are more effective in the dentin structure, which has a higher water content, than in tooth enamel [23]. However, on the other hand it has been reported that laser use eliminates smear layer formation, allowing bonding and restorative materials to be applied directly to the tooth surface without the need for etching with acid [24].Treating the enamel surface with an erbium laser induces irregularities, fostering enhanced micromechanical retention and allowing adhesive penetration [20]. Consequently, the formation of resin tags is promoted, augmenting micromechanical adhesion. Morphological electron microscopic examinations showed an improvement in micromechanical retention following laser irradiation due to increased surface roughness and enlargement of the enamel-resin interface [25]. Considering these findings, laser conditioning was preferred in the current study as a suitable alternative to selective phosphoric acid etching. While some studies have shown that surfaces treated with erbium lasers have similar micro-irregularities to those treated with phosphoric acid [26], many other studies [27, 28] have found that laser etching results in lower bonding effectiveness than acid etching before applying bonding agents in direct adhesive restorations. Microleakage assessments in the current study remarkably revealed that the laser-conditioning groups (Group 5 and 6) exhibited greater leakage in both the occlusal and gingival regions compared to Group 1 and Group 3. This result shows that the etching process to increase bonding in enamel with low water content cannot be achieved only with a laser. In addition, apart from the negative conditions of the enamel, the variable intensity per unit area resulting from manual laser application and the natural difficulty in controlling the time at the edges of the enamel are among the factors that affect this result. It is conceivable that additional conditioning with phosphoric acid after laser etching may positively affect the bond strength and decrease leakage. This deficiency in determining the groups could be identified as the primary limitation of this study.

Although universal adhesive systems have shown promise, the Clearfil SE bond, which incorporates 10-methacryolyloxydecyl dihydrogen phosphate (10-MDP) functional monomer, remains the gold standard in self-etch adhesive systems [29]. The chemical bonding between 10-MDP in the adhesive system and calcium (Ca) within the hydroxyapatite structure forms a hybrid layer that is more resistant to hydrolytic degradation. This is due to the presence of the chemical bonding between these two components [30, 31]. Regardless of the adhesive system used, a stable hybrid layer is essential for ensuring the long-term clinical success of restoration [32]. Although there are limited microleakage studies on the self-curing Tokuyama Universal Bond, it has been reported in the literature to exhibit higher microleakage values compared to various universal adhesive systems, particularly in comparison to Clearfil SE Bond [32, 33]. Based on the evidence of previous studies, it can be inferred that the 10-MDP monomer, which is present in the composition of Clearfil SE Bond, played a role in slightly lower microleakage values. In a study conducted by Kibe et al. [34], the marginal adaptation of self-cured and light-cured universal adhesive systems in Class V cavities was examined, and results demonstrated a significantly higher marginal adaptation with the use of self-cured universal adhesive. Conversely, in the current study, Tokuyama Universal Bond, a self-cured universal adhesive system, demonstrated slightly high or significantly higher values of microleakage regardless of the conditioning method employed, as opposed to Clearfil SE Bond, a light-cured adhesive [34]. It is possible that the cavity depth played a role in these outcomes, given that there is an interaction between cavity depth, microleakage values, and the performance of light-cured adhesives [35]. In the present investigation, the comparable values observed between the self-cured adhesive system Tokuyama Universal Bond and the light-cured Clearfil SE Bond across the majority of groups may be ascribed to the standardized cavity depth of 2 mm. This particular depth facilitates an effective polymerization depth within the light-cured adhesive system. The rejection of the second null hypothesis further supports this conclusion.

The thermocycling procedure induces contraction/expansion stresses and hastened chemical degradation, replicating the clinical environment to which the restorations are subjected [36]. A previous study stated that thermal aging impacted the development of gaps at the adhesive interface, irrespective of the restorative material used [37]. In this present study, the thermocycling protocol was employed to simulate long-term bonding efficacy. It was reported that the majority of studies evaluated in the in-vitro study conducted by Dietschi et al. [38] did not test microleakage by subjecting it to mechanical loading and thermal cycling. Evaluations conducted without functional stress can only assess the resistance of the tooth-restoration interface to leakage based on the physicochemical properties of the materials and restorative technique used. In our study, only the thermal cycling method was employed for aging without applying functional stress, which constitutes another limitation of our study.

Various techniques, including the use of different dyes, chemical markers, radioactive isotopes, and air pressure, have been employed to assess the extent of microleakage and the dependability of restorations at the margins [39]. The dye diffusion method is widely employed and among the most frequently utilized techniques. Commonly used dyes include methylen blue, basic fucsin, eosin, and aniline blue [40]. Methylene blue has a small molecular weight, making it easy to apply. Furthermore, it is straightforward to use and its cost is relatively low [41]. The major drawbacks of this method are the inability to quantitatively assess the extent of leakage across the entire tooth-restoration interface and the subjective scoring of the extent of dye penetration by operators with a specified range of values [42]. The ImageJ software was employed to mitigate the inherent limitations of visual scoring, facilitating a semi-quantitative assessment of the acquired data in millimeters (mm). The correlation analyses in the present study disclosed a strong association between the measurements in millimeters obtained through the ImageJ program and the scores attributed to images captured by the light microscope. This underscores the suitability of both methodologies for microleakage studies.

The light microscope and SEM are widely used in microleakage studies at the dentin bonding interface [21]. According to a previous study, the results of SEM and light microscopy analysis were similar, and SEM evaluations improved microleakage analysis [43]. With this context, through SEM imaging, enamel edges treated in laser groups exhibited micro-irregularities, in the present study. SEM micromorphologic analyses are recognized for their enhanced reliability in providing detailed and accurate results [44]. Within the constraints of laboratory investigations, quantitative marginal analysis using SEM has been documented as a precise and dependable method for assessing marginal adaptation [45]. Additionally, the light microscope, being a more cost-effective alternative, can yield microleakage observations comparable to SEM [44]. The images acquired through both scanning electron microscopy (SEM) and light microscopy underwent a two-dimensional and semi-quantitative evaluation. The limitation in assessing the sealing abilities of adhesive systems with different conditioning protocols in three dimensions constitutes the final constraint in the present study.

The pre-clinical in-vitro assessment of adhesive systems and restorative materials holds significant importance, particularly in evaluating the success of dental restorations and adhesive systems interface concerning marginal compatibility. Hence, an in-vitro environment was preferred to compare the microleakage effectiveness of light-cured two-step self-etch and self-cured universal adhesive systems in class V cavities selectively etched with phosphoric acid or Er, Cr: YSGG lasers. However, it is acknowledged that in vitro experiments may not entirely replicate clinical conditions. Therefore, further clinical studies should be designed to evaluate the microleakage levels of different adhesive systems in varying application modes.

## Conclusion

Based on the results of this study and taking into account the limitations of an in vitro examination, the occlusal margins demonstrated better outcomes than the gingival margins, regardless of the type of adhesive system used or the enamel etching techniques employed. Clearfil SE Bond has been demonstrated to outperform Tokuyama Universal Bond adhesive system, regardless of the etching protocol utilized. The etching enamel margins with phosphoric acid could improve the performance of self-eth adhesive system, while laser conditioning increased the success of the Universal adhesive system. A high correlation was established between the measurements obtained using the Image J program and the scores assigned to the images obtained from the light microscope in the study of microleakage. This suggests that both methods could be considered suitable for microleakage studies.

## Supplementary information

Below is the link to the electronic supplementary material.

ESM 1 (DOCX 30.1 KB)

ESM 2 (DOCX 16.8 KB)

## Data Availability

The datasets and materials used or analysed during the current study are available from the corresponding author on reasonable request.

## Declarations

This study was performed in line with the principles of the Declarations of Helsinki. The study protocol was approved by Clinical Research Ethics Committee of Nuh Naci Yazgan University (no.2023/004–006).
