# Supplementary material for: Impact of acid and laser etching of enamel on microleakage in different adhesive systems
Source: Lasers Med Sci. 2024 Jul 15;39(1):181. doi: 10.1007/s10103-024-04120-0 (PMC11249556; doi:10.1007/s10103-024-04120-0)
Supplement: Supplementary file 1 — (DOCX 30.1 KB) [file 10103_2024_4120_MOESM1_ESM.docx]

| **Supp. Table 1. Results of comparison of microleakage scores in occlusal and enamel gingival sites within the groups** | | | | | | | | | |
| --- | --- | --- | --- | --- | --- | --- | --- | --- | --- |
|  |  | **Treatment Groups** | | | | | | Test İs. | p* |
| **Assesment methods** | **Tooth region** | **Group 1** | **Group 2**  **No-conditioning** | **Group 3** | **Group 4**  **Selective Acid Etching** | **Group 5** | **Group 6**  **Selective Laser Etching** |  |  |
| **Image J** | Occlusal | 0.16 ± 0.25 | 0.27 ± 0.49 | 0.08 ± 0.18 | 0.27 ± 0.27 | 0.49 ± 0.38 | 0.21 ± 0.17 | 37.84 | **<0.001*** |
|  |  | 0.00 (0.00 – 0.81) ^ab^ | 0.00 (0.00 – 1.35) ^bd^ | 0.00 (0.00 – 0.65) ^a^ | 0.26 (0.00 – 0.80) ^bd^ | 0.46 (0.00 - 1.45)^cd^ | 0.21 (0.00 – 0.60) ^abd^ |  |  |
|  | Gingival | 0.47 ± 0.56 | 0.70 ± 0.44 | 0.33 ± 0.49 | 0.27 ± 0.30 | 0.95 ± 0.33 | 0.54 ± 0.28 | 30.19 | **<0.001*** |
|  |  | 0.16 (0.00 – 1.44) ^a^ | 0.77 (0.00 – 1.47) ^b^ | 0.00 (0.00 – 1.14) ^a^ | 0.18 (0.00 – 0.97) ^a^ | 0.90 (0.33 – 1.54) ^b^ | 0.62 (0.00 – 0.93) ^ab^ |  |  |
| Test İst. | | -1.804 | -2.487 | -1.836 | -0.199 | -3.98 | -3.136 |  |  |
| p**/*** | | 0.071** | **0.013**** | 0.066** | 0.842** | **0.001***** | **0.002**** |  |  |
| **Examiner 1** | Occlusal | 0.42 ± 0.51 | 0.68 ± 1.16 | 0.32 ± 0.67 | 0.84 ± 0.69 | 1.32 ± 0.95 | 0.84 ± 0.50 | 27.23 | **<0.001*** |
|  |  | 0.00 (0.00 – 1.00) ^ab^ | 0.00 (0.00 – 3.00) ^b^ | 0.00 (0.00 – 2.00) ^a^ | 1.00 (0.00 – 2.00) ^bc^ | 1.00 (0.00 – 3.00) ^c^ | 1.00 (0.00 – 2.00) ^bc^ |  |  |
|  | Gingival | 1.21 ± 1.27 | 1.26 ± 0.81 | 0.84 ± 1.26 | 0.95 ± 1.03 | 2.47 ± 0.61 | 1.53 ± 0.70 | 29.58 | **<0.001*** |
|  |  | 1.00 (0.00 – 3.00) ^ab^ | 1.00 (0.00 – 3.00) ^ab^ | 0.00 (0.00 – 3.00) ^ab^ | 1.00 (0.00 – 3.00) ^ab^ | 3.00 (1.00 – 3.00) ^c^ | 2.00 (0.00 – 2.00) ^bc^ |  |  |
| Test İst. | | -2.218 | -1.6 | -1.628 | -0.318 | -3.054 | -2.968 |  | |
| p** | | **0.027**** | 0.110** | 0.103** | 0.751** | **0.002**** | **0.003**** |  |  |
| **Examiner 2** | Occlusal | 0.47 ± 0.70 | 0.74 ± 1.24 | 0.32 ± 0.67 | 0.68 ± 0.82 | 1.58 ± 1.02 | 1.05 ± 0.62 | 34.66 | **<0.001*** |
|  |  | 0.00 (0.00 – 2.00) ^ab^ | 0.00 (0.00 – 3.00) ^ab^ | 0.00 (0.00 – 2.00) ^a^ | 0.00 (0.00 – 2.00) ^ab^ | 2.00 (0.00 – 3.00) ^c^ | 1.00 (0.00 – 2.00) ^bc^ |  |  |
|  | Gingival | 1.21 ± 1.36 | 1.74 ± 0.99 | 0.84 ± 1.26 | 1.32 ± 1.16 | 2.89 ± 0.32 | 2.26 ± 1.10 | 36.16 | **<0.001*** |
|  |  | 1.00 (0.00 – 3.00) ^ab^ | 2.00 (0.00 – 3.00) ^b^ | 0.00 (0.00 – 3.00) ^a^ | 1.00 (0.00 – 3.00) ^ab^ | 3.00 (2.00 – 3.00) ^c^ | 3.00 (0.00 – 3.00) ^bc^ |  |  |
| Test İst. | | -1.969 | -2.364 | -1.628 | -2.029 | -3.36 | -3.348 |  |  |
| p** | | **0.049**** | **0.018**** | 0.103** | **0.042**** | **0.001**** | **0.001**** |  |  |
| Group 1: No condition + Light-cured SE; Group 2: No condition + self-cured Universal; Group 3: Selective Acid Etching + Light-cured SE; Group 4: Selective Acid Etching + Self-cured Universal; Group 5: Laser + Light-cured SE; Group 6: Laser + Self-cured Universal.  *Kruskal Wallis Test; Mean ± standard deviation; Median (minimum-maximum); a-d: No difference exists between groups with the same letter.  ** Wilcoxon Test; ***Paired Two Sample t Test; Mean ± standard deviation; Median (minimum-maximum) | | | | | | | | | |

**IMPACT OF ACID AND LASER ETCHING OF ENAMEL ON MICROLEAKAGE IN DIFFERENT ADHESIVE SYSTEMS**

Lasers in Medical Science

**Atılan Yavuz Sevim^1^, Erturk Avunduk Ayse Tugba^1^, Karatas Ozcan^2^, Çakır Kılınç Nazire Nurdan^2^, Delikan Ebru^3^**

**Corresponding Author:** Sevim ATILAN YAVUZ, Mersin University, Faculty of Dentistry, dtsevimatilan@gmail.com
