# Supplementary material for: Impact of acid and laser etching of enamel on microleakage in different adhesive systems
Source: Lasers Med Sci. 2024 Jul 15;39(1):181. doi: 10.1007/s10103-024-04120-0 (PMC11249556; doi:10.1007/s10103-024-04120-0)
Supplement: Supplementary file 2 — (DOCX 16.8 KB) [file 10103_2024_4120_MOESM2_ESM.docx]

**Supp. Table 2. Examination of inter-observer agreement and agreement between Image J and visual measurements**

|  | ICC(%95 CI) | p |
| --- | --- | --- |
| Examiner 1 – Examiner 2 Occlusal | 0,907 (0,865 - 0,936) | **<0,001** |
| Examiner 1 – Examiner 2 Gingival | 0,954 (0,933 - 0,968) | **<0,001** |
| Image J- Examiner 1 Occlusal | 0,794 (0,701 - 0,858) | **<0,001** |
| Image J - Examiner 1 Gingival | 0,781 (0,682 - 0,848) | **<0,001** |
| Image J - Examiner 2 Occlusal | 0,725 (0,602 - 0,81) | **<0,001** |
| Image J - Examiner 2 Gingival | 0,713 (0,584 - 0,802) | **<0,001** |

CI: Correlation Coefficient (95%), ICC: Interclass coefficient corelation

**IMPACT OF ACID AND LASER ETCHING OF ENAMEL ON MICROLEAKAGE IN DIFFERENT ADHESIVE SYSTEMS**

Lasers in Medical Science

**Atılan Yavuz Sevim^1^, Erturk Avunduk Ayse Tugba^1^, Karatas Ozcan^2^, Çakır Kılınç Nazire Nurdan^2^, Delikan Ebru^3^**

**Corresponding Author:** Sevim ATILAN YAVUZ, Mersin University, Faculty of Dentistry, dtsevimatilan@gmail.com
